# Insights into the impacts of autotoxic allelochemicals from rhizosphere of *Atractylodes lancea* on soil microenvironments

**DOI:** 10.3389/fpls.2023.1136833

**Published:** 2023-03-10

**Authors:** Meng Wang, Juan Deng, Gonghao Duan, Lei Chen, Xiao Huang, Wenjie Wang, Ling Gong, Yan Zhang, Kun Yu, Lanping Guo

**Affiliations:** ^1^ College of Pharmacy, Hubei University of Chinese Medicine, Wuhan, China; ^2^ Hubei Key Laboratory of Resources and Chemistry of Chinese Medicine, Hubei University of Chinese Medicine, Wuhan, China; ^3^ State Key Laboratory of Dao-di Herbs, National Resource Center for Chinese Materia Medica, China Academy of Chinese Medical Sciences, Beijing, China

**Keywords:** allelochemicals, *Atractylodes lancea*, microbial community, natural fallow, root exudates

## Abstract

*Atractylodes lancea* suffers from continuous cropping obstacles that have become a major constraint in its cultivation, but there is still little information on the autotoxic allelochemicals and their interaction with soil microorganisms. In this study, we firstly identified the autotoxic allelochemicals from rhizosphere of *A. lancea* and determined their autotoxicity. Third-year continuous *A. lancea* cropping soils, i.e., rhizospheric soil and bulk soil, compared with control soil and one-year natural fallow soil were used to determine soil biochemical properties and microbial community. Eight allelochemicals from *A. lancea* roots were detected and exhibited significant autotoxicity effects on seed germination and seedling growth of *A. lancea* with the highest content of dibutyl phthalate in rhizospheric soil and lowest IC_50_ value of 2,4-di-tert-butylphenol inhibiting seed germination. The contents of soil nutrients and organic matter, pH value, and enzyme activity were altered between different soils, and the parameters of fallow soil were close to those of the unplanted soil. The PCoA analysis indicated that the community composition of both bacteria and fungi were differed significantly among the soil samples. Continuous cropping decreased OTUs numbers of bacterial and fungal communities, and natural fallow restored them. The relative abundance of Proteobacteria, Planctomycetes, and Actinobacteria decreased, and that of Acidobacteria and Ascomycota increased after three years cultivation. The LEfSe analysis identified 115 and 49 biomarkers for bacterial and fungal communities, respectively. The results suggested that natural fallow restored the structure of soil microbial community. Overall, our results revealed that autotoxic allelochemicals caused the variations of soil microenvironments and resulted in replantation problem of *A. lancea*, and natural fallow alleviated the soil deterioration by remodeling the rhizospheric microbial community and restoring soil biochemical properties. These findings provide important insights and clues for solving the continuous cropping problems and guiding the management of sustainable farmland.

## 1 Introduction

The continuous planting of a single crop or its related species on the same field results in a reduction in crop yield and quality (Arafat et al., 2020), which is often connected to the variation of the microbiome and reduction in the abundance of beneficial microbes (Wu et al., 2018). Chen et al. (2019) found that the practice of consecutive monoculture facilitated the enrichment of rhizosphere pathogenic microorganisms and eventually led to the emergence of replant disease in *Rehmannia glutinosa*. Chen et al. (2021) revealed that the microbial community structure and diversity varied dramatically in the rhizosphere soils of *Atractylodes lancea* after 3 years’ cultivation. The consecutive monoculture of sweet potato (*Ipomoea batatas*) led to an imbalance in the population of soil microbes resulting in the enrichment of pathogenic bacteria and a reduction in beneficial bacteria (Li et al., 2019a). The variety of genes that are differentially regulated under a consecutive monoculture practice indicated the potential important roles of the soil microbiome in the growth of *R. glutinosa* (Tian et al., 2017) and green chiretta (*Andrographis paniculate*) (Li et al., 2019b). However, the mechanisms that underly the modification of plant-soil interactions by the microbial communities remain unclear.

Root exudates are also closely related to the consecutive monoculture problem. These exudates typically consist of a diverse set of primary metabolites, such as amino acids, sugars, and carboxylic acids among others, and secondary metabolites that are critically involved in soil ecosystem functions (Min et al., 2015), such as signaling molecules, stimulants, or repellents (Baetz and Martinoia, 2014). The autotoxicity of root exudates is one of the primary reasons for the consecutive monoculture problem in cucumber (*Cucumis sativus*) (Xiao et al., 2020a). Phytotoxic metabolites released from roots are important reasons for the replant problem in *R. glutinosa* (Zhang et al., 2019). The allelopathic potential of watermelon (*Citrullus lanatus*) plays an important role in “soil sickness” (Hao et al., 2007). High concentrations of cinnamic acid and vanillin inhibit the growth of eggplant and even cause verticillium wilt, which is one of the reasons for eggplant continuous cropping obstacles (Chen et al., 2011). Allelopathic substances, such as palmitic acid and phthalic acid, were identified in the root exudates of *Lilium lanzhouensis* with serious continuous cropping obstacles (Wu et al., 2015). The root exudates of soybean monocropped for 13 years reduced rhizosphere nitrification and changed soil microbial community (Wang et al., 2012). Microbial communities are reported to be affected by root exudates (Zhao et al., 2021), which can restrict the recovery of soil functions (Yan et al., 2021).

Natural fallow has been identified as a conventional practice used worldwide to overcome the obstacles of continuous planting. Fallow lands tend to naturally replenish their soil fertility and characteristics. Lintemani et al. (2020) suggested a 15-year fallow after a crop cycle to restore the original soil conditions in slash-and-burn agriculture. The rhizosphere bacteria and fungi of early colonizing annual plants are considered to accelerate the process of improving soil fertility and potentially boost the succession of vegetation during fallow periods (Hauchhum and Tripathi, 2019). However, our knowledge on the changes of soil microenvironment under natural fallow practices remains unclear.

*Atractylodes lancea* (Thunb.) DC., a member of the Asteraceae family, is a perennial herb in eastern Asia that is mostly used to treat digestive disorders, rheumatic diseases, and night blindness among other ailments. The plant is typically 30 to 100 cm height. The leaves are elliptic to lanceolate with spinulose margin, and the lower and middle ones sometimes divide pinnately into 3-7 segments. The capitula is composed of white tubular florets. Modern pharmacological studies showed the broad pharmacological effects of the *A. lancea* rhizome on cancers, inflammation, nervous, gastrointestinal, and cardiovascular systems (Koonrungsesomboon et al., 2014) owing to its abundant active substances, such as sesquiterpenoids and polyacetylene glycosides (Zhang et al., 2021). The rhizomes of *A. lancea* are also important raw material of nutraceutical and cosmetic industries for the essential oils. In recent years, *A. lancea* has been an important economic crop that has attracted increasing attention, and it is cultivated on more than 10,000 ha in China. Continuous cropping obstacles are one of the most severe hindrances of *A. lancea* cultivation, which substantially reduce the yield and quality of the rhizomes and cause severe soilborne diseases. Guo et al. (2006) reported the autotoxicity of root exudates associated with the continuous cropping of *A. lancea*. However, the allelopathic compounds in root exudates have not yet been identified in *A. lancea*.

The alteration of rhizosphere microbiome was reported in consecutive monocultures and may result in the susceptibility of *A. lancea* to diseases (Guo et al., 2007). However, there is little information on the continuous cropping obstacles in *A. lancea* and the specific mechanism of the recovery of fallow lands. The aims of this study were to: (i) identify the autotoxic allelochemicals from rhizosphere and determined their autotoxicity; (ii) evaluate changes in the soil microenvironment after continuous monoculture with *A. lancea*; and (iii) explore the potential mechanism of natural fallow on the recovery of soil microenvironment.

## 2 Materials and methods

### 2.1 Plant material, growth conditions, and sampling

The *A. lancea* plants were grown in the fields at the experimental station located in Yingshan, Hubei Province of China (30°58′N, 115°56′E) with the same soil type and topography using normal cultivation methods, including fertilization and field management practices. The site is characterized by a subtropical humid monsoon climate, with an average annual rainfall of approximately 1400 mm and an average annual temperature of 16.4°C. The regional natural soil belongs to yellow brown soil according to Chinese soil classification system. After three years’ continuously grown (P3), the soil that strongly adhered to the roots of *A. lancea* were sampled as the rhizospheric soil (P3R), and the remaining soil as bulk soil (P3B). Both the rhizospheric and bulk soil samples with three replicates were collected. In another field, the plants were planted for two years in the same manner as P3, harvested, and fallowed naturally for one year (F). The field with no crop grown was used as control (P0). Weeding was not performed at site F and P0, and three most abundant weeds were *Erigeron canadensis*, *Ageratum conyzoides*, and *Imperata cylindrica*. Three soil samples were randomly selected from the plow layer in F and P0. Soil samples were collected on September 12, 2020. The soil samples used to detect the soil microorganisms were placed in sterile PVC tubes, immediately frozen in liquid nitrogen, and stored at -80°C. The other soil samples were placed in sterile bags and stored at 4°C.

### 2.2 Gas chromatography-mass spectrometry analysis

A total of 100 g of soil samples of the four treatments were extracted in 100 mL of methanol followed by evaporation to 1 mL in a 35°C water bath. The extracts were then filtered through a polytetrafluoroethylene syringe filter (0.22 μm), and 100 μl of the filtered sample was added to the internal standard naphthalene. The sample was analyzed using a Thermo ISQ QD-TRACE 1300 GC-MS (Thermo Fisher, Waltham, MA, USA) as described by Zheng et al. (2018). The relative concentrations of the eight compounds with the highest peak area in the soil were calculated by comparison with the peak area of the internal standard.

### 2.3 Pot experiment

The pot experiment was conducted in a 25°C greenhouse at 65% relative humidity under a 12 h light/12 h dark photoperiod. The *A. lancea* seeds were sown evenly into polycarbonate pots (20 cm × 15 cm × 10 cm) with 30 seeds per pot. The pots with 3 replicates were watered with 20 ml water or 2 g/ml rhizosphere soil extracts (RE), and the rates of germination were recorded.

### 2.4 Single substance experiment

The identified autotoxic allelochemicals, i.e., 2,4-di-tert-butylphenol, methyl palmitate, dibutyl phthalate, 7,9-di-tert-butyl-1-oxaspiro [4.5] deca-6,9-diene-2,8-dione, 2,2’-methylenebis, palmitic acid, methyl salicylate, and methyl stearate, with different concentrations were employed to test for their autotoxcity using *A. lancea* seeds. Thirty *A. lancea* seeds were sown evenly in each petri plate with 30 seeds per plate for each treatment and replicated three times respectively. The half-maximal inhibitory concentrations (IC_50_) of the compounds were determined by using probit analysis.

### 2.5 Determination of soil properties

The soil was air-dried naturally, passed through a 0.2 mm sieve, and then used to detect soil enzyme activities and chemical analyses. The contents of soil organic carbon (SOC), alkaline hydrolysis of nitrogen (AN), available phosphorus (AP) and quick-acting potassium (QK) were measured using the Chinese national standards NY 1121.6-2006, LY/T 1228-2015, NY/T 1121.7-2014, and NY/T 889-2004, respectively. The pH from the soil water suspension (1:5 [w/v]) was measured using a pH meter (FE 20). The activity of urease and sucrase was determined colorimetrically by using a spectrophotometer (UVmini-1240; Shimadzu, Kyoto, Japan) as previously described (Li et al., 2021a).

### 2.6 16S rRNA and ITS gene sequencing

DNA was extracted using HiPure Soil DNA Kits (Magen, Guangzhou, China), and the 16S rRNA genes were amplified using specific primers 341F (5′-CCTACGGGNGGCWGCAG-3′), 806R (5′-GGACTACHVGGGTATCTAAT-3′); ITS3-KYO2 (5′-GATGAAGAACGYAGYRAA-3′), ITS4 (5′-TCCTCCGCTTATTGATATGC-3′) (Cao et al., 2023). PCR amplicons were quantified using an ABI Step One Plus Real-Time PCR System (Life Technologies, Foster City, USA). The purified amplicons were pooled and sequenced on an Illumina platform (PE250, San Diego, CA, USA).

### 2.7 Statistical analysis

The data were analyzed by SPSS 19.0 (IBM, Armonk, NY, USA) and GraphPad Prism 8.0 (San Diego, CA, USA). One-way analysis of variance followed by the least significance difference test was used when appropriate. Differences were considered significant when the *p*-value was < 0.05. The abundance-based coverage (ACE) and Shannon indices were calculated in QIIME. Multivariate statistical techniques, including a principal coordinates analysis (PCoA) and the biomarker features in each group were screened by LEfSe software.

## 3 Results

### 3.1 Identification and variation of autotoxic allelochemicals

**Figure 1:**
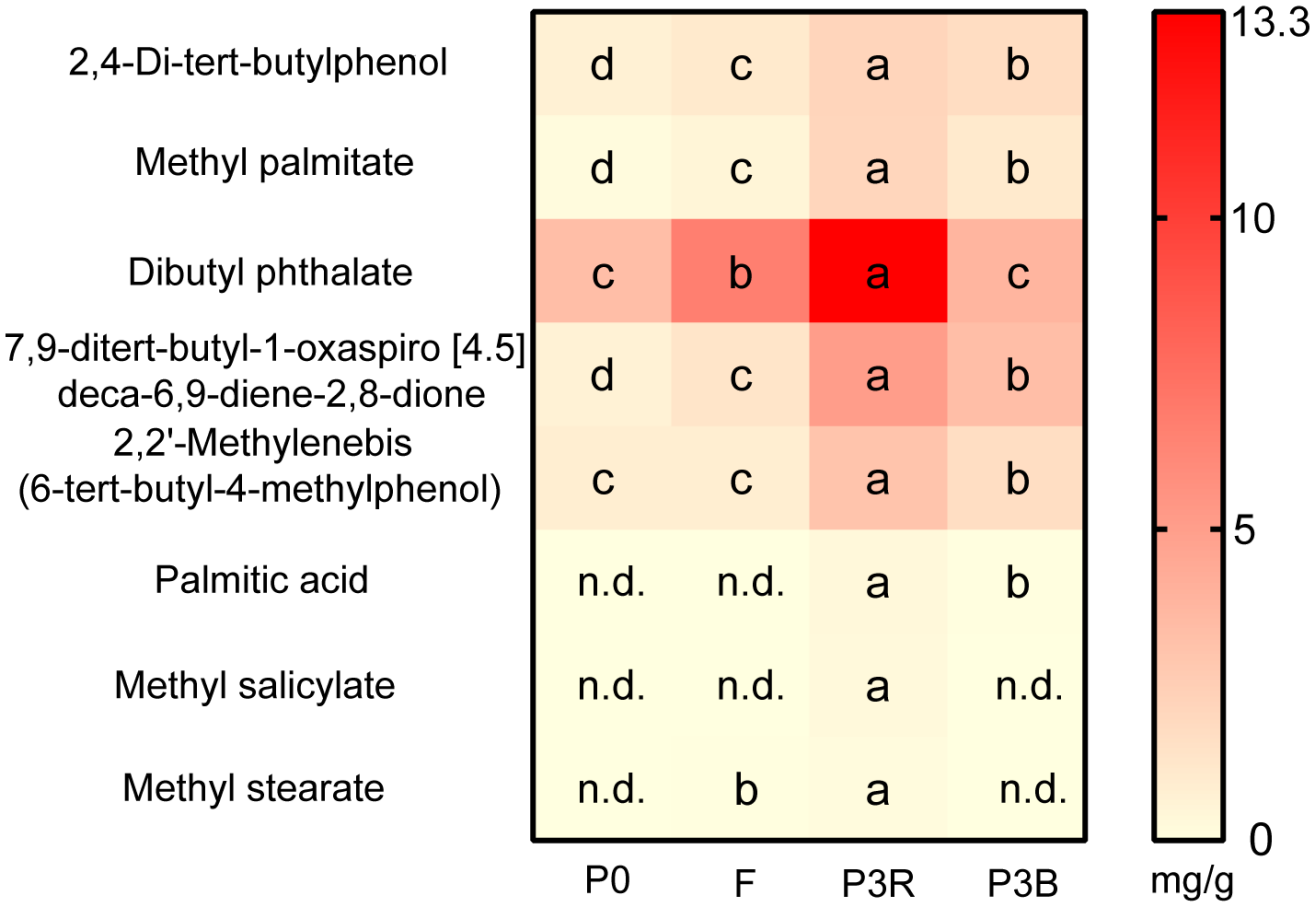
Relative concentrations of eight toxic compounds in the soil. Values represent means ± SD (n = 3). Different letters indicate significant differences according to LSD test (p < 0.05), n.d., not detected. P0, control soil; F, one-year fallow soil; P3R, Rhizosphere soil after three years consecutive monoculture; P3B, Bulk soil after three years consecutive monoculture.

Eight autotoxic substances from *A. lancea* roots were detected by GC-MS with the similarity more than 80%, and dibutyl phthalate has the highest content in the P3R soil (up to 13.3 mg/g) (**Figure 1**). In the F treatment group, the contents of these eight compounds were lower than those in the P3R and P3B treatments but were higher than those in the P0 treatment. This indicates that the root exudates in the soil were gradually degraded or disappeared over time after fallow.

**Figure 2:**
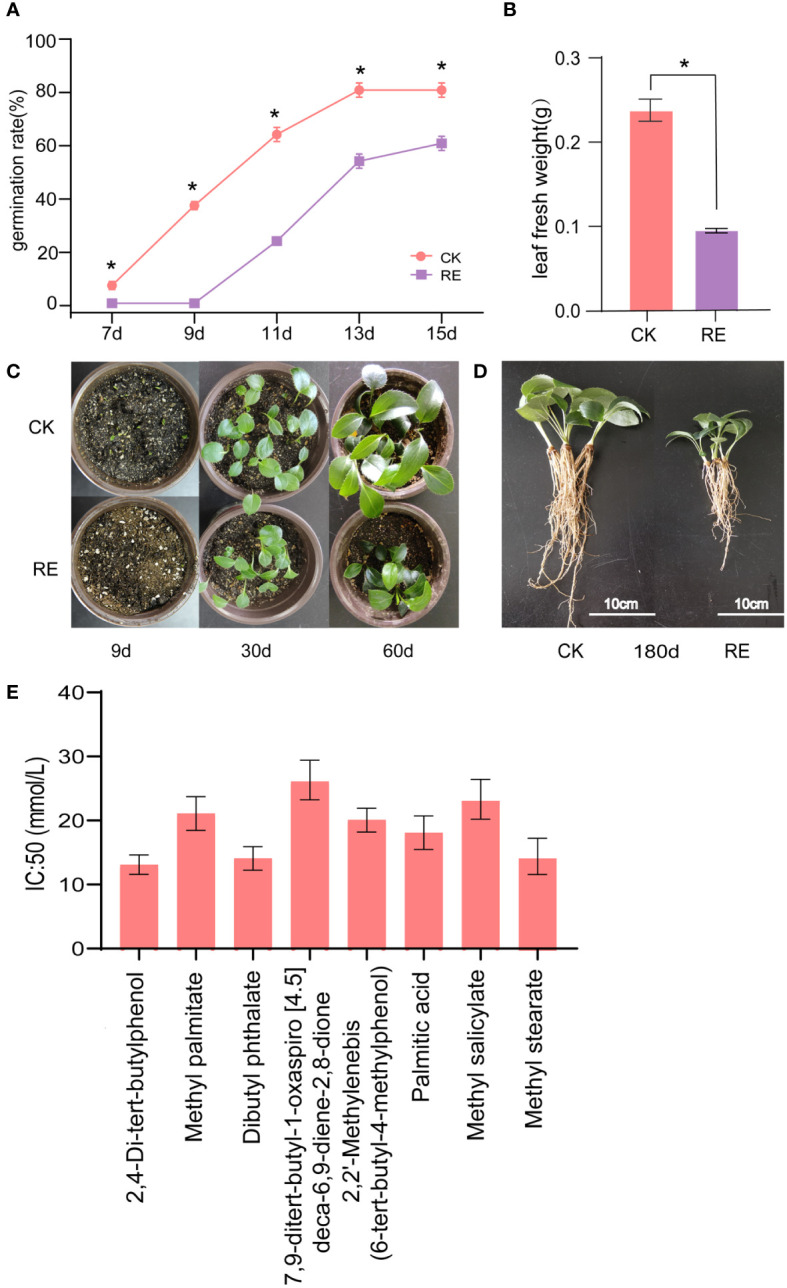
Autotoxic effects of the rhizosphere soil extracts of *A lancea*. **(A)** Seed germination rate. **(B)** Leaf fresh weight. **(C)** Growth morphology of *A lancea* at day 9, 30, and 60 after sowing. **(D)** *A lancea* seedling on day 180. Values represent means ± SD (n = 3 replicates with 30 plants). **(E)** The half-maximal inhibitory concentrations (IC_50_) of the compounds. *, significant difference between the CK and RE (p < 0.05). CK, control; RE, rhizosphere soil extracts.

The rhizosphere soil extracts of *A. lancea* showed autotoxicity during seed germination and seedling growth. The germination rates and leaf weights were significant lower in the RE treatment with the decrement rate of 25.08% and 65.38%, respectively, suggesting that the rhizosphere soil extracts are autotoxic (**Figure 2A, B**). The growth of *A. lancea* seedlings on day 9, 30, 60, and 180 further supported this finding (**Figure 2C, D**).

The single substance experiments were carried out to further elucidate the toxicity of the identified autotoxic allelochemicals. The inhibition in germination of *A. lancea* seeds expressed in IC_50_ are shown in **Figure 2E**. 2,4-di-tert-butylphenol had the lowest IC_50_ value (13.05 mol/L) followed by dibutyl phthalate (14.08 mol/L) and methyl stearate (14.12 mol/L).

### 3.2 Changes in the soil biochemical properties

The contents of soil nutrients and organic matter, pH value, and enzyme activity were used to evaluate the soil biochemical properties. The contents of AP in P3R and P3B were significantly higher than those of P0. The contents of QK in P3R and AN in P3B were significantly different than those of P0. The irregular change of soil nutrients could be owing to fertilization during the cultivation of *A. lancea.* The pH values were significantly decreased up to 8% in P3R or P3B compared with those in P0, and the same tendencies could be seen in SOC content and urease activity, while the sucrase activity increased. The parameters of F were between those of the unplanted and continuous cropping soil. It is worth noting that the activities of sucrase and urease in P3R changed more dramatically than those in P3B (**Table 1**).

**Table 1:** Contents of AN, AP, QK, SOC, and pH value, sucrase activity, and urease activity.

	AP (mg/Kg)	AN (mg/Kg)	QK (mg/Kg)	Soc (g/Kg)	pH	Sucrase (g/Kg)	Urease (g/Kg)
P0	9.30 ± 1.99c	61.04 ± 0.55b	183.36 ± 7.37b	19.06 ± 0.33b	6.03 ± 0.03a	0.31 ± 0.03d	9.94 ± 0.28a
F	28.36 ± 1.53b	64.51 ± 0.51a	179.4 ± 2.061b	21.85 ± 0.47a	5.96 ± 0.05a	0.42 ± 0.25c	9.92 ± 0.17a
P3R	69.19 ± 3.11a	60.59 ± 0.71b	334.64 ± 6.14a	15.9 ± 0.55c	5.85 ± 0.05b	0.54 ± 0.02a	7.17 ± 0.21c
P3B	29.89 ± 0.82b	58.14 ± 0.64c	205.24 ± 5.12b	4.25 ± 0.45d	5.53 ± 0.04c	0.43 ± 0.01b	9.26 ± 0.16b

Different letters indicate significant differences according to LSD test (p < 0.05). AN, alkaline hydrolysis of nitrogen; AP, available phosphorus; QK, quick-acting potassium; SOC, soil organic carbon. Values represent means ± SD (n = 3).

### 3.3 Modulation of soil microorganisms

#### 3.3.1 Microbial community richness and diversity

There was no significant difference between P0 and F on the diversity of fungi and bacteria based on the Shannon indices. For the Ace indices, significant differences were observed only between P3R and P3B. The results of group F tended to P0, suggesting that natural fallow alleviated the results (**Table 2**).

**Table 2:** Alpha diversity indices.

Alpha	Bacteria		Fungi	
	Shannon index	Ace index	Shannon index	Ace index
P0	9.78 ± 0.33a	5,891.14 ± 217.29a	4.14 ± 0.28a	301.83 ± 16.69ab
F	9.55 ± 0.22a	5,728.69 ± 130.32a	3.33 ± 0.41a	294.59 ± 9.34ab
P3R	9.44 ± 0.16a	4,679.35 ± 470.45b	3.70 ± 0.23a	309.15 ± 8.98a
P3B	9.84 ± 0.10a	6,386.82 ± 14.34a	3.99 ± 0.16a	271.41 ± 8.94b

Different letters represent significant differences (p < 0.05). Values are means ± SD (n = 3). ANOVA, analysis of variance; LSD, least significant difference; SD, standard deviation.

#### 3.3.2 Microbial community composition

**Figure 3:**
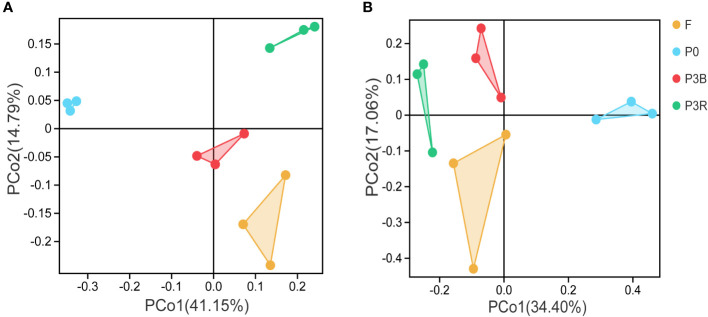
PCoA of microbial communities at the phylum level. **(A)** Bacteria; **(B)** Fungi. P0, control soil; F, one-year fallow soil; P3R, Rhizosphere soil after three years consecutive monoculture; P3B, Bulk soil after three years consecutive monoculture.

The distinct differences were shown in the microbial community structure among bacteria and fungi at the phylum level (**Figure 3**). The first two principal components (PC1 and PC2) of the PCoA in bacteria accounted for 41.15% and 14.79% of the total variation, respectively (**Figure 3A**), while they accounted for 34.40% and 17.06% of the total variation in fungi, respectively (**Figure 3B**).

The PCoA analysis indicated that the community composition of both bacteria and fungi were differed significantly among the soil samples, which suggested that the bacterial and fungal structures changed markedly during the cultivation of *A. lancea*.

#### 3.3.3 Specific microbial clades

**Figure 4:**
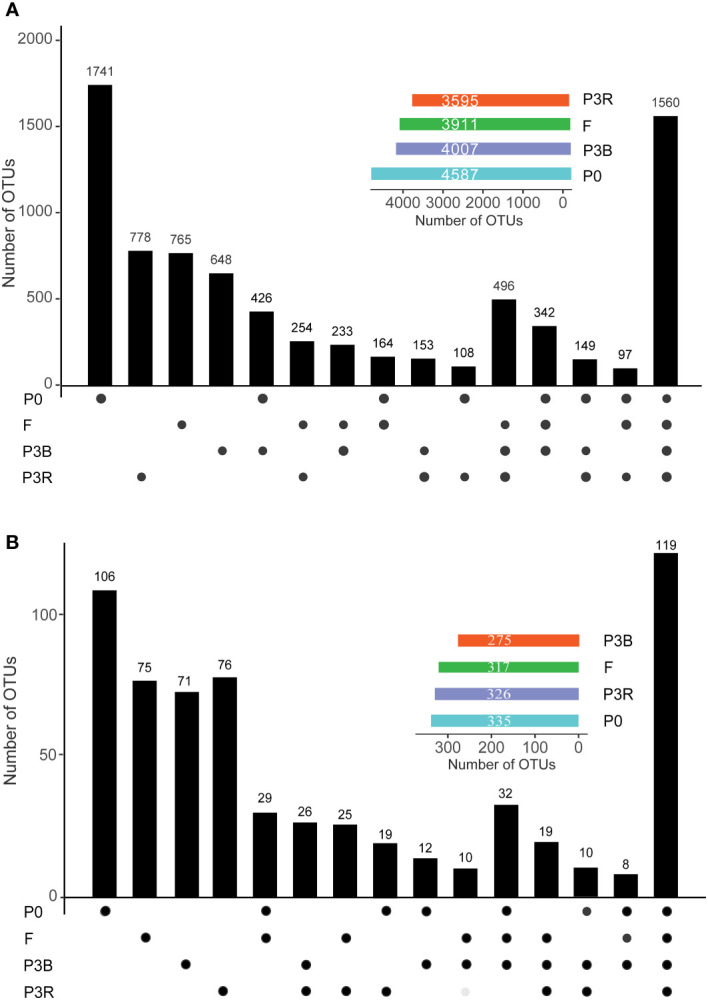
Number of unique and shared OTUs in each treatment group. **(A)** Bacteria; **(B)** Fungi. Insets show total number of OTUs in each group. OTUs, operational taxonomic units. P0, control soil; F, one-year fallow soil; P3R, Rhizosphere soil after three years consecutive monoculture; P3B, Bulk soil after three years consecutive monoculture.

A total of 1,560 bacterial (**Figure 4A**) and 119 fungi (**Figure 4B**) operational taxonomic units (OTUs) were consistently present in all samples. The lowest number of OTUs was observed in P3R and P3B for the bacterial and fungal communities (**Figure 4B**), respectively, indicating that continuous planting led to variation in the rhizosphere microorganisms. The number of OTUs in F was higher than those in P3R and P3B, suggesting that natural fallow restored the structure of soil microbial community (**Figure 4**).

**Figure 5:**
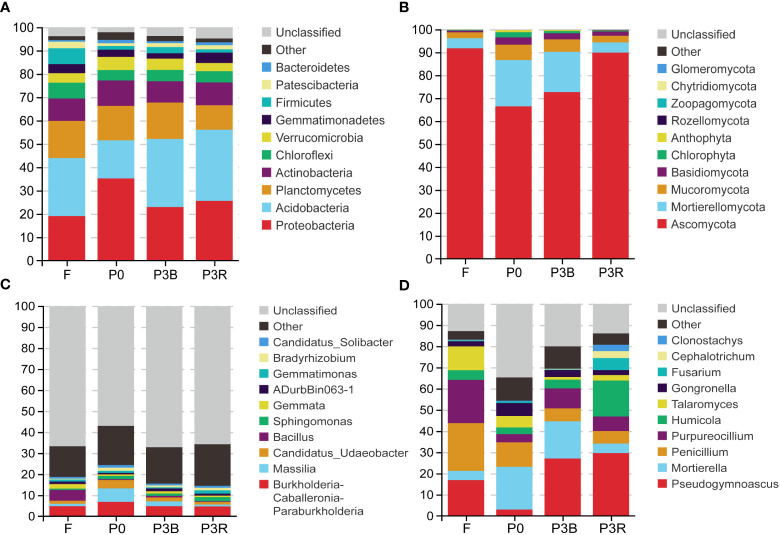
Relative abundances of the top 10 microbial phylum and genus levels. **(A, C)** Bacteria; **(B, D)** Fungi;**(A, B)** phylum levels; **(C, D)** genus levels. P0, control soil; F, one-year fallow soil; P3R, Rhizosphere soil after three years consecutive monoculture; P3B, Bulk soil after three years consecutive monoculture.

The relative abundance of different phyla and genera in different soils is shown in **Figures 5A, C**. Acidobacteria, Proteobacteria, Planctomycetes and Actinobacteria were the dominant flora, accounting for more than 65% of the entire bacterial microbial flora (**Figure 5A**). The relative abundance of Proteobacteria, Planctomycetes and Actinobacteria was decreased, and that of Acidobacteria increased after three years of cultivation of *A. lancea*, resulting in a shift of the highest relative abundance of Proteobacteria to that of Acidobacteria. Besides, the relative abundance of Chloroflexi, Firmicutes and Patescibacteria in the F soil was the highest in four soil groups. At the genus level, the relative abundance of *Burkholderia-Caballeronia-Paraburkholderia* and *Massilia* decreased during the cultivation of *A. lancea* (**Figure 5C**).

The phylum Ascomycota largely dominated the soil fungal communities and accounted for 66.42-91.75% of all fungi found in this study. The relative abundance of Ascomycota increased significantly after three years of cultivation. (**Figure 5B**) Notably, cultivation influenced fungal enrichment in the soil. For example, in the three years rhizosphere soil, the *Fusarium* genus accounted for the largest proportion at 5.7%, but it was less abundant (0.28–0.75%) in other soils. Genus *Pseudogymnoascus* accounted for the largest proportion at 29.61% in the P3R treatment, but it accouned for least proportion at 2.97% in P0 soil (**Figure 5D**).

**Figure 6:**
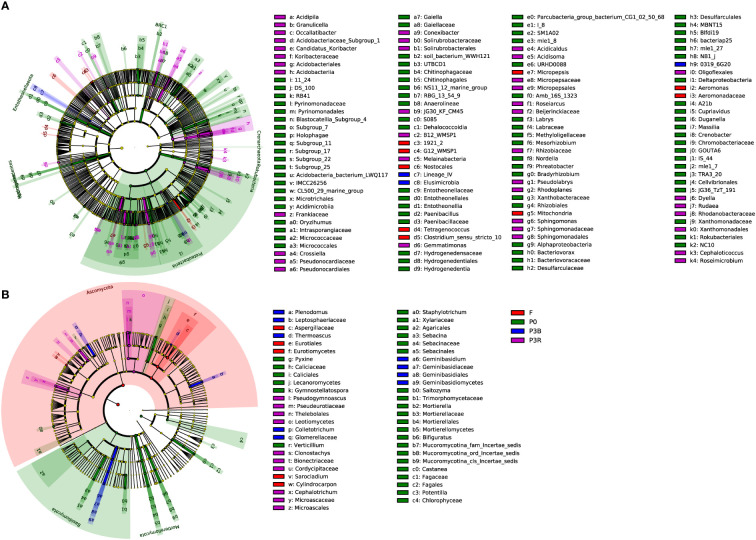
Histogram and cladogram based on the LEfSe analysis of soil microbial communities. **(A)** Bacteria; **(B)** Fungi. Indicator bacteria with LDA scores of 2.5 or greater. P0, control soil; F, one-year fallow soil; P3R, Rhizosphere soil after three years consecutive monoculture; P3B, Bulk soil after three years consecutive monoculture.

A total of 115 biomarkers were identified using LEfSe analysis (LDA values > 2.5) for bacterial communities, which showed that the significant changes of the abundance of different groups were related to different farming methods (**Figure 6A**). Four bacterial taxa were observed in F soil, and the most important contribution was from 1921_2 (at the genus level). Twelve bacterial taxa were observed in the P0 soil, and the most important contribution was from Proteobacteria (at the phylum level). Only one bacterial taxa was observed in P3B soil, and the most important contribution was from Lineage_IV (at the order level). Seven bacterial taxa were observed in the P3R treatment, and the most important contribution was from Acidobacteriia (at the class level).

The LEfSe analysis also identified 49 biomarkers for fungal communities (LDA values > 2.5) (**Figure 6B**). In the F soil, the important contribution at the order level was from Eurotiales (Ascomycota). In the P0 soil, the important contributions were from Mortierella (at the genus level). In the P3B soil, two groups of fungi (Ascomycota and Basidiomycota) were significantly enriched at the phylum level, and the important contributions were from Mortierella (at the genus level). In the P3R treatment, the important contribution at the genus level was from genus *Pseudogymnoascus*.

### 3.4 Obtained model for autotoxic allelochemicals

**Figure 7:**
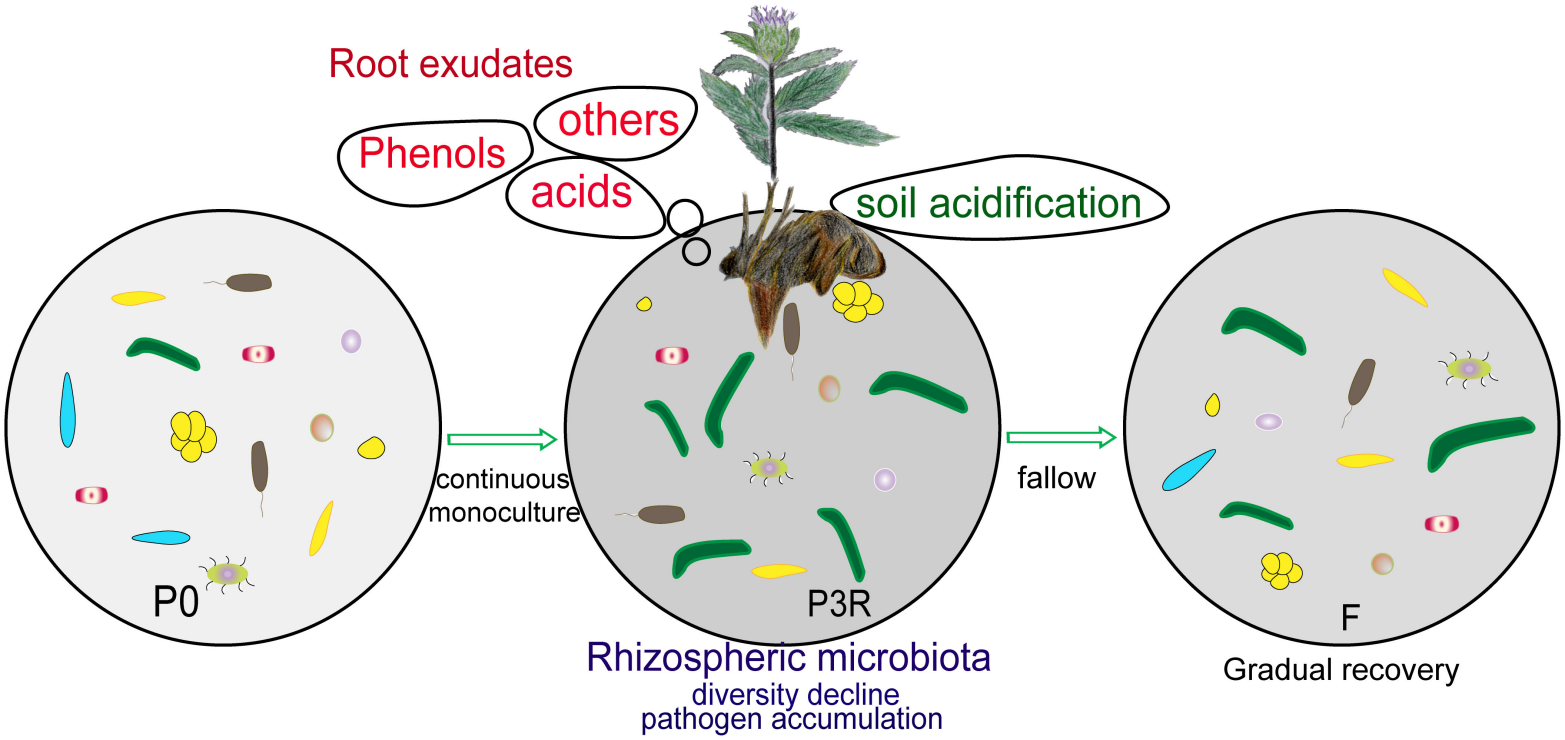
Schematics of natural fallow alleviating the deterioration of soil caused by the continuous monoculture of *A. lancea*. P0, control soil; F, one-year fallow soil; P3R, Rhizosphere soil after three years consecutive monoculture.

Based on the above analysis, we obtain the following model for autotoxic allelochemicals in *A. lancea* (**Figure 7**). The autotoxic allelochemicals (dibutyl phthalate, 2,4-di-tert-butylphenol, etc.) released from plant roots lead to soil acidification, the enrichment of pathogen (such as *Fusarium* spp.), and decline of microbiota diversity, which result in the deterioration of soil microenvironments, and then cause the replantation problem. Whereas natural fallow restored soil deterioration by degrading autotoxic allelochemicals, improving soil physicochemical properties, and remolding microbial communities.

## 4 Discussion

The continuous monoculture technique results in the inhibition of plant growth and reduces yield and quality, which is reported to be closely related to the accumulation of root exudates. A series of chemical substances, such as 2,4-di-tert-butylphenol, palmitic acid and dibutyl phthalate among others, were identified in the rhizosphere of *A. lancea* and inhibited the germination of seeds and growth of seedlings. In our results, after three years of continuous cropping, the content of dibutyl phthalate in the rhizosphere soil of *A. lancea* was the highest. Dibutyl phthalate has also been identified as an autotoxic substance of Lanzhou lily and cucumber, which has a significant inhibitory effect on the seedling growth and changes the soil microbial community (Yan et al., 2019; Li et al., 2021b). 2,4-di-tert-butylphenol was found to be an autotoxic compound that exhibited strong allelopathic effects in *A. macrocephala*, and inhibited microbial growth and promoted the increase of genus *Fusarium* at a particular concentration in Lanzhou lily (Zheng et al., 2018; Cui et al., 2022), which is in agreement with our results in *A. lancea*. With the accumulation of this compound, the numbers of pathogenic microorganisms increased, resulting in the obstacles of continuous cropping. Replant conditions caused a significant decrease in vegetative growth (Henfrey et al., 2015), which is consistent with our results. Therefore 2,4-di-tert-butylphenol and dibutyl phthalate were the main autotoxic allelochemicals in *A. lancea* due to their relatively high content and autotoxicity in the rhizosphere.

The soil chemical properties are reported to control the kinetics of soil enzymes (Tan et al., 2021). Consecutive cropping was frequently found to result in soil acidification and an imbalance in soil nutrients. Although the fertilizer regime applied to the fields interfered with the soil nutrients, the pH value, SOC content and activity of soil enzymes differed significantly after three years of *A. lancea* cultivation. Most of the eight compounds identified from the rhizosphere soil are acids, which were closely related to the enhancement of soil acidity in continuous cropping. Implementing a natural fallow alleviated the problems with these soil factors. Similar results were obtained by Hauchhum and Tripathi (Hauchhum and Tripathi, 2019). Thus, the autotoxic substances exuded from *A. lancea* roots accumulated in the soil and resulted in soil acidification, which in turn inhibited plant growth and initiated the continuous cropping obstacle of *A. lancea*.

The plant root is the interface between plant and soil, which is the richest microbial ecosystem on earth (Bulgarelli et al., 2012). Our results on the composition and concentration of root exudates can shape the microbial community structure in the rhizosphere soil (Li et al., 2013; Xiao et al., 2020b). Our results on the composition and the relative concentration of root exudates in *A. lancea* also support this concept. Studies have found that Actinobacteria serve as biofertilizers in the formulation of agricultural products (Saidi et al., 2021). The reduced proportion of Actinomycetes in this study indicated the unfavorable soil microbial environment for the growth of *A. lancea*. Many plant beneficial *Pseudomonas* species have been confirmed to function as plant growth promoting rhizobacteria (PGPR) (Zboralski and Filion, 2020). The relative abundance of Ascomycota and Mortierellomycota was higher in diseased and healthy soils, respectively (Yuan et al., 2020). Basidiomycota can form symbioses with plants to form mycorrhizae, which benefit the cultivation of crops. Numerous studies have indicated that *Fusarium solani* can cause damage to plant roots (Luo et al., 2014). Our results of the microbial community structure in *A. lancea* are not only consistent with these concepts but also indicate the vicious cycle among accumulated root exudates and an imbalanced microbial community. In our study, the indicator species in unplanted soil are the most. With continuous planting, the rhizosphere indicator species decreased in the third year, but the rhizosphere indicator material is more than the non-rhizosphere group, which indicates that the root has a certain aggregation effect on the microbial community. Plants can recruit and enrich specialized PGPR from the soil, such as *Bacillus subtilis* L2 for pepper (Samaniego-Gámez et al., 2016) and Arbuscular Mycorrhizal Fungi (AMF) for wheat (*Triticum aestivum* L.) (Yadav et al., 2020). Despite this, we demonstrated that the effects of beneficial bacteria could not reverse the harm of an imbalanced microbial community caused by the accumulation of allelochemicals in the rhizosphere of *A. lancea*.

It has been reported that the soil pH can drive the composition of microbial community (Liu et al., 2020). Soil pH was one of the dominant limiting factors indirectly affecting bacterial diversity (Duan et al., 2022). In our study, the altered root exudate profiles and rebound of the pH value correlated with the increase in microbial community richness and diversity in fallow soil compared with that in the soil of consecutive cropping. Firmicutes are known to be a dominant bacterial group (Filippidou et al., 2015). The relative abundance of Firmicutes in the F soil was the highest in our study, indicating that the soil microbial environment had self-corrected, which is consistent with the findings of other studies (Guo et al., 2006; Hauchhum and Tripathi, 2019; Lintemani et al., 2020). In addition, after fallow, the numbers of harmful bacteria decreased, while those of the beneficial bacteria gradually increased, which indicated that the microbial community repaired itself after fallow.

## 5 Conclusion

In summary, we have shown that allelochemical compounds secreted from *A. lancea* roots modulate plant performance by altering soil physicochemical environment and rhizosphere microbial communities, and natural fallow alleviates the deterioration of soil by improving soil microbial communities. Our results have important implications for solving the continuous cropping problems of *A. lancea* and guiding sustainable farmland management. Further studies are merited to determine the periods of natural fallow and explore intercropping practice to shorten the fallow periods.

## Data availability statement

The data is publicly accessible at: https://ngdc.cncb.ac.cn/gsa, accession number CRA009378, CRA009379.

## Author contributions

KY and LPG conceived and designed the research. MW and JD performed the experiments, analyzed the results and wrote the manuscript. GD, LC, XH, WW, LG, and YZ analyzed and interpreted the data. All authors contributed to the article and approved the submitted version.

## References

[B1] Arafat Y. Din I. U. Tayyab M. Jiang Y. Chen T. Cai Z.. (2020). Soil sickness in aged tea plantation is associated with a shift in microbial communities as a result of plant polyphenol accumulation in the tea gardens. Front. Plant Sci. 11. doi: 10.3389/fpls.2020.00601 PMC7270330 32547573

[B2] Baetz U. Martinoia E. (2014). Root exudates: The hidden part of plant defense. Trends Plant Sci. 2, 90–98. doi: 10.1016/j.tplants.2013.11.006 24332225

[B3] Bulgarelli D. Rott M. Schlaeppi K. Themaat E. V. L. Ahmadinejad N. Assenza F.. (2012). Revealing structure and assembly cues for arabidopsis root-inhabiting bacterial microbiota. Nature 488, 91–95. doi: 10.1038/nature11336 22859207

[B4] Cao Y. Du P. Yin B. Zhou S. Li Z. Zhang X.. (2023). Melatonin and dopamine enhance waterlogging tolerance by modulating ROS scavenging, nitrogen uptake, and the rhizosphere microbial community in *Malus hupehensis* . Plant Soil 483, 475–493. doi: 10.1007/s11104-022-05759-w

[B5] Chen A. Gu L. Xu N. Feng F. Chen D. Yang C.. (2019). NB-LRRs not responding consecutively to *Fusarium oxysporum* proliferation caused replant disease formation of *Rehmannia glutinosa* . Int. J. Mol. Sci. 213, 3203. doi: 10.3390/ijms20133203 PMC6651281 31261891

[B6] Chen L. Wu X. Xu Y. Wang B. Liu S. Niu J.. (2021). Microbial diversity and community structure changes in the rhizosphere soils of *Atractylodes lancea* from different planting years. Plant Signal. Behav. 2, e1854507. doi: 10.1080/15592324.2020.1854507 PMC7849755 33289592

[B7] Chen S. Zhou B. Lin S. Li X. Ye X. (2011). Accumulation of cinnamic acid and vanillin in eggplant root exudates and the relationship with continuous cropping obstacle. Afr. J. Biotechnol. 10, 2659–2665. doi: 10.5897/AJB10.1338

[B8] Cui J. Zhang E. Zhang X. Wang Q. Liu Q. (2022). Effects of 2,4-di-tert-butylphenol at different concentrations on soil functionality and microbial community structure in the lanzhou lily rhizosphere. appl. Soil Ecol. 172, 104367. doi: 10.1016/j.apsoil.2021.104367

[B9] Duan N. Li L. Liang X. McDearis R. Fine A. K. Cheng Z.. (2022). Composition of soil viral and bacterial communities after long-term tillage, fertilization, and cover cropping management. Appl. Soil Ecol. 177, 104510. doi: 10.1016/j.apsoil.2022.104510

[B10] Filippidou S. Junier T. Wunderlin T. Lo C. Li P. Chain P. S.. (2015). Under-detection of endospore-forming firmicutes in metagenomic data. Comput. Struct. Biotechnol. J. 13, 299–306. doi: 10.1016/j.csbj.2015.04.002 25973144 PMC4427659

[B11] Guo L. Huang L. Jiang Y. Chen M. Lv D. Zeng Y. (2007). Change of microbial community in rhizoma sphere of cultivated *Atractylodes lancea* . China J. Chin. Mater. Med. 32, 1131–1133. doi: 10.3321/j.issn:1001-5302.2007.12.003 17802868

[B12] Guo L. Huang L. Jiang Y. Chen B. Zhu Y. Zeng Y.. (2006). Bioactivity of extracts from rhizoma and rhizosphere soil of cultivated *Atractylodes lancea* DC. and identification of their allelopathic compounds. J. Ecol. 26, 528–535. doi: 10.3321/j.issn:1000-0933.2006.02.028

[B13] Hao Z. P. Wang Q. Christie P. Li X. L. (2007). Allelopathic potential of watermelon tissues and root exudates. Sci. Hortic. 112, 315–320. doi: 10.1016/j.scienta.2006.12.030

[B14] Hauchhum R. Tripathi S. K. (2019). Impact of rhizosphere microbes of three early colonizing annual plants on improving soil fertility during vegetation establishment under different fallow periods following shifting cultivation. Agric. Res. 9, 213–221. doi: 10.1007/s40003-019-00422-w

[B15] Henfrey J. L. Baab G. Schmitz M. (2015). Physiological stress responses in apple under replant conditions. Sci. Hortic. 194, 111–117. doi: 10.1016/j.scienta.2015.07.034

[B16] Koonrungsesomboon N. Na-bangchang K. Karbwang J. (2014). Therapeutic potential and pharmacological activities of *Atractylodes lancea* (Thunb.) DC. Asian Pac. J. Trop. Med. 7, 421–428. doi: 10.1016/S1995-7645(14)60069-9 25066389

[B17] Li J. Chen X. Zhan R. He R. (2019b). Transcriptome profiling reveals metabolic alteration in *Andrographis paniculata* in response to continuous cropping. Ind. Crop Prod 137, 585–596. doi: 10.1016/j.indcrop.2019.05.067

[B18] Li S. Li Z. Feng X. Zhou F. Wang J. Li Y. (2021a). Effects of biochar additions on the soil chemical properties, bacterial community structure and rape growth in an acid purple soil. Plant Soil Environ. 67, 121–129. doi: 10.17221/390/2020-PSE

[B19] Li S. Wang L. Li Y. Huang F. Yu H. Zhang Y.. (2021b). Biodegradation of di-n-butyl phthalate in rhizosphere and growth-promoting effect of *Cucumis sativus linn.* by a novel pseudomonas sp. DNB-S1. Ecotoxicol. 30, 1454–1464. doi: 10.1007/s10646-020-02287-0 33094413

[B20] Li H. Wang J. Liu Q. Zhou Z. Chen F. Xiang D. (2019a). Effects of consecutive monoculture of sweet potato on soil bacterial community as determined by pyrosequencing. J. Basic. Microbiol. 59, 181–191. doi: 10.1002/jobm.201800304 30288775

[B21] Li X. Xia Z. Kong C. Xu X. (2013). Mobility and microbial activity of allelochemicals in soil. J. Agric. Food Chem. 61, 5072–5079. doi: 10.1021/jf400949m 23647315

[B22] Lintemani M. G. Loss A. Mendes C. S. Fantini A. C. (2020). Long fallows allow soil regeneration in slash-and-burn agriculture. J. Sci. Food Agric. 100, 1142–1154. doi: 10.1002/jsfa.10123 31680261

[B23] Liu T. Wu X. Li. H. Alharbi H. Wang J. Dang P.. (2020). Soil organic matter, nitrogen and pH driven change in bacterial community following forest conversion. For. Ecol. Manage. 477, 118473. doi: 10.1016/j.foreco.2020.118473

[B24] Luo X. Li J. Dong J. Sui A. Sheng M. Yang X. (2014). First report of *Fusarium solani* causing root rot on *Coptis chinensis* in southwestern china. Plant Dis. 98, 1273. doi: 10.1094/PDIS-02-14-0164-PDN 30699657

[B25] Min K. Freeman C. Kang H. Choi S. (2015). The regulation by phenolic compounds of soil organic matter dynamics under a changing environment. BioMed. Res. Int. 2015, 825098. doi: 10.1155/2015/825098 26495314 PMC4606107

[B26] Saidi S. Cherif-Silini H. Bouket A. C. Silini A. Eshelli M. Luptakova L.. (2021). Improvement of *Medicago sativa* crops productivity by the co-inoculation of *Sinorhizobium meliloti*-actinobacteria under salt stress. Curr. Microbiol. 78, 1344–1357. doi: 10.1007/s00284-021-02394-z 33646380 PMC7997840

[B27] Samaniego-Gámez B. Y. Garruña R. Tun-suárez J. M. Kantun-Can J. Reyes-Ramirez A. Cervantes-Diaz L. (2016). Bacillus spp. inoculation improves photosystem II efficiency and enhances photosynthesis in pepper plants. Chil. J. Agric. Res. 76, 409–416. doi: 10.4067/S0718-58392016000400003

[B28] Tan X. Nie Y. Ma X. Guo Z. Liu Y. Tian H.. (2021). Soil chemical properties rather than the abundance of active and potentially active microorganisms control soil enzyme kinetics. Sci. Total Environ. 770, 144500. doi: 10.1016/j.scitotenv.2020.144500 33736358

[B29] Tian Y. Feng F. Zhang B. Li M. Wang F. Gu L.. (2017). Transcriptome analysis reveals metabolic alteration due to consecutive monoculture and abiotic stress stimuli in *Rehamannia glutinosa* libosch. Plant Cell Rep. 36, 859–875. doi: 10.1007/s00299-017-2115-2 28275853

[B30] Wang J. Li X. Zhang J. Tao T. Wei D. Wang Y.. (2012). Effect of root exudates on beneficial microorganisms–evidence from a continuous soybean monoculture. Plant Ecol. 213, 1883–1892. doi: 10.2307/23362488

[B31] Wu L. Chen J. Xiao Z. G. Zhu X. C. Wang J. Y. Wu H.. (2018). Barcoded pyrosequencing reveals a shift in the bacterial community in the rhizosphere and rhizoplane of *Rehmannia glutinosa* under consecutive monoculture. Int. J. Mol. Sci. 19, 850. doi: 10.3390/ijms19030850 29538311 PMC5877711

[B32] Wu Z. J. Xie Z. K. Yang L. Wang R. Y. Guo Z. H. Zhang Y. B.. (2015). Identification of autotoxins from root exudates of lanzhou lily (*Lilium davidii* var. *unicolor*). Allelopathy J. 35, 35–48.

[B33] Xiao X. Lv J. Xie J. Feng Z. Ma N. Li J.. (2020a). Transcriptome analysis reveals the different response to toxic stress in rootstock grafted and non-grafted cucumber seedlings. Int. J. Mol. Sci. 21, 774. doi: 10.3390/ijms21030774 31991638 PMC7037640

[B34] Xiao Z. Zou T. Lu S. Xu Z. (2020b). Soil microorganisms interacting with residue-derived allelochemicals effects on seed germination. Saudi. J. Biol. Sci. 27, 1057–1065. doi: 10.1016/j.sjbs.2020.01.013 32256166 PMC7105660

[B35] Yadav R. Ror P. Rathore P. Ramakrishna W. (2020). Bacteria from native soil in combination with arbuscular mycorrhizal fungi augment wheat yield and biofortification. Plant Physiol. Biochem. 150, 222–233. doi: 10.1016/j.plaphy.2020.02.039 32155450

[B36] Yan Z. He X. Guo K. Li X. Yang X. Jin H.. (2019). Allelochemicals from the rhizosphere of lanzhou lily: Discovery of the autotoxic compounds of a bulb crop. Sci. Hortic. 250, 121–126. doi: 10.1016/j.scienta.2019.02.038

[B37] Yan M. Li T. Li X. Liu Y. Zhang G. (2021). Microbial biomass and activity restrict soil function recovery of a post-mining land in eastern loess plateau. Catena 199, 105107. doi: 10.1016/j.catena.2020.105107

[B38] Yuan J. Wen T. Zhang H. Zhao M. Penton C. R. Thomashow L. S.. (2020). Predicting disease occurrence with high accuracy based on soil macroecological patterns of fusarium wilt. ISME J. 14, 2936–2950. doi: 10.1038/s41396-020-0720-5 32681158 PMC7784920

[B39] Zboralski A. Filion M. (2020). Genetic factors involved in rhizosphere colonization by phytobeneficial *Pseudomonas* spp. Comput. Struct. Bioetchnol. J. 18, 3539–3554. doi: 10.1016/j.csbj.2020.11.025 PMC7711191 33304453

[B40] Zhang B. Weston P. A. Gu L. Zhang B. Li M. Wang F.. (2019). Identification of phytotoxic metabolites released from *Rehmannia glutinosa* suggest their importance in the formation of its replant problem. Plant Soil 441, 439–454. doi: 10.1007/s11104-019-04136-4

[B41] Zhang W. Zhao Z. Chang L. Cao Y. Wang S. Kang C.. (2021). Atractylodis rhizoma: A review of its traditional uses, phytochemistry, pharmacology, toxicology and quality control. J. Ethnopharmacol. 266, 113415. doi: 10.1016/j.jep.2020.113415 32987126 PMC7521906

[B42] Zhao M. Zhao J. Yuan J. Hale L. Wen T. Huang Q.. (2021). Root exudates drive soil-microbe-nutrient feedbacks in response to plant growth. Plant Cell Environ. 44, 613–628. doi: 10.1111/pce.13928 33103781

[B43] Zheng F. Chen L. Gao J. Niu F. Duan X. Yin L.. (2018). Identification of autotoxic compounds from *Atractylodes macrocephala* koidz and preliminary investigations of their influences on immune system. J. Plant Physiol. 230, 33–39. doi: 10.1016/j.jplph.2018.08.006 30144693

